# A randomised controlled trial of positive memory training for the treatment of depression within schizophrenia

**DOI:** 10.1186/s12888-015-0453-6

**Published:** 2015-04-14

**Authors:** Craig Steel, Mark van der Gaag, Kees Korrelboom, Judit Simon, Peter Phiri, M Fazil Baksh, Til Wykes, Diana Rose, Suzanna Rose, Mark Hardcastle, Simon Enright, Gareth Evans, David Kingdon

**Affiliations:** School of Psychology and Clinical Language Sciences, University of Reading, Reading, RG6 6AL UK; VU University and EMGO Institute for Health and Care Research, van der Boechorststraat 1, 1081 BT Amsterdam, The Netherlands; PsyQ, Parnassia-Bavo Psychiatric Centre, Stadhoudersplantsoen 2, 2517 JL The Hague, The Netherlands; Department of Health Economics, Centre for Public Health, Medical University of Vienna, Vienna, Austria; Southern Health NHS Foundation Trust, Southampton, SO30 3JB UK; School of Mathematical and Physical Sciences, University of Reading, Reading, RG6 6AL UK; Department of Psychology, Institute of Psychiatry, King’s College London, De Crespigny Park, London, SE5 8AF UK; Health Service Research Department, Institute of Psychiatry, King’s College London, De Crespigny Park, London, SE5 8AF UK; Berkshire Healthcare Foundation Trust, Bracknell, RG12 ILH UK; Department of Medicine, University of Southampton, Southampton, SO17 1BJ UK

## Abstract

**Background:**

Depression is highly prevalent within individuals diagnosed with schizophrenia, and is associated with an increased risk of suicide. There are no current evidence based treatments for low mood within this group. The specific targeting of co-morbid conditions within complex mental health problems lends itself to the development of short-term structured interventions which are relatively easy to disseminate within health services. A brief cognitive intervention based on a competitive memory theory of depression, is being evaluated in terms of its effectiveness in reducing depression within this group.

**Methods/Design:**

This is a single blind, intention-to-treat, multi-site, randomized controlled trial comparing Positive Memory Training plus Treatment as Usual with Treatment as Usual alone. Participants will be recruited from two NHS Trusts in Southern England. In order to be eligible, participants must have a DSM-V diagnosis of schizophrenia or schizo-affective disorder and exhibit at least a mild level of depression. Following baseline assessment eligible participants will be randomly allocated to either the Positive Memory Training plus Treatment as Usual group or the Treatment as Usual group. Outcome will be assessed at the end of treatment (3-months) and at 6-month and 9-month post randomization by assessors blind to group allocation. The primary outcome will be levels of depression and secondary outcomes will be severity of psychotic symptoms and cost-effectiveness. Semi-structured interviews will be conducted with all participants who are allocated to the treatment group so as to explore the acceptability of the intervention.

**Discussion:**

Cognitive behaviour therapy is recommended for individuals diagnosed with schizophrenia. However, the number of sessions and length of training required to deliver this intervention has caused a limit in availability. The current trial will evaluate a short-term structured protocol which targets a co-morbid condition often considered of primary importance by service users. If successful the intervention will be an important addition to current initiatives aimed at increasing access to psychological therapies for people diagnosed with severe mental health problems.

**Trial registration:**

Current Controlled Trials. ISRCTN99485756. Registered 13 March 2014.

## Background

The annual cost of the treatment of schizophrenia within the UK has been estimated at over £2billion, which is approximately 3% of the NHS budget [1]. Consequently, the development of cost-effective interventions for schizophrenia is a priority. Current UK government guidelines recommend psychological therapy for the treatment of schizophrenia. Specifically, cognitive-behavioural therapy (CBT), in the form of at least 16 sessions over a minimum of 6 months, is advised [2]. Most current evidence-based CBT protocols for the treatment of schizophrenia are designed to be generic and flexible. This enables the clinician to work with the wide range of complex presentations, including high levels of co-morbidity, that are associated with a diagnosis of schizophrenia. However, it is often a co-morbid diagnosis such as posttraumatic stress disorder or depression that both the clinician and patient consider the priority for intervention.

About half of the people diagnosed with schizophrenia also suffer from depression [3]. This co-morbid condition is associated with particularly high levels of health care use, and a suicide rate of 5% [4]. The effectiveness of anti-depressants is equivocal [5] in this group. To date, there have been no clinical trials to evaluate the effectiveness of a psychological intervention which targets the treatment of depression within these co-morbid patients. The evidence base that supports psychological interventions for depression, such as CBT [6], behavioural activation [7] and mindfulness [8] is derived from trials which have excluded individuals diagnosed with schizophrenia. There is some evidence that 6 to 12 months of generic CBT targeting the symptoms of schizophrenia does achieve a small to moderate effect on mood [9]. However, the training that is required for NHS clinicians to provide this generic intervention is lengthy, expensive, and limited to a relatively small number of specialists. Consequently, there is limited access to CBT for people diagnosed with schizophrenia. The targeting of conditions co-morbid with schizophrenia, such as depression, lends itself to the development of short-term structured psychological interventions, which can be packaged into the form of a manualised protocol. These interventions are relatively easy to disseminate within health services, and can be used by a wide range of clinicians. They are also likely to be cost-effective.

An enhanced therapeutic effect may be obtained through the use of interventions which specifically target the cognitive processes associated with mood disorder within this group. A recent theoretical account of mood disorder [10] suggests that individuals simultaneously hold both positive and negative self-representations. However, depression is associated with heightened access to the negative self-representations, with the relevant pathway being strengthened through regular activation. Korrelboom and colleagues have developed an intervention which consists of a training programme specifically designed to heighten the activation of positive self-representations. The intervention, termed competitive memory training (CoMeT), has been shown to be effective in reducing levels of depression and increasing self-esteem within a range of different disorders [11-15]. Within one study, a mediation analysis suggested that the reduction in depression was mediated by self-esteem [15]. An adapted form of this intervention termed positive memory training (PoMeT) has been developed for use within a U.K. based trial. The PoMeT trial is the first randomised controlled trial to specifically target the treatment of depression in people diagnosed with schizophrenia.

### Hypotheses

The primary hypotheses to be tested is whether within patients who are diagnosed with schizophrenia and exhibit at least a mild level of depression, those who receive positive memory training will demonstrate a higher level of reduction in depressive symptoms than those who receive treatment as usual.

Secondary hypotheses predict that:

- Positive memory training will reduce the level of distressing psychotic symptoms, as measured by the Psychotic Symptom Rating Scale [16].
- Positive memory training will be cost-effective.

## Method

The trial is funded by the National Institute of Health Research and received ethical approval for all sites from the Berkshire Research Ethics Committee (REC ref 13/SC/0634). The RCT is conducted by a multidisciplinary team of researchers, clinicians, statisticians and health economists within a number of European institutions and NHS Trusts within the South of England.

## Design

**Figure 1:**
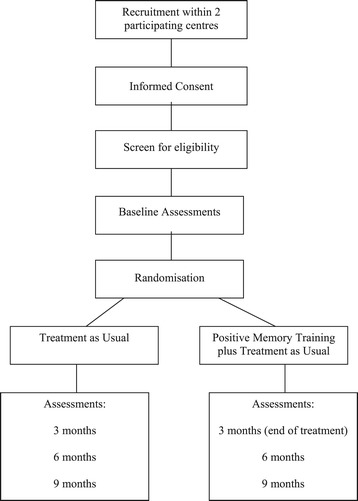
Flow chart of study design.

A rater-blind randomised controlled trial using intention to treat comparing positive memory training (PoMeT) and treatment as usual (TAU) with TAU alone. The intervention is to be completed within 3-months from the date of randomisation. Assessments are conducted at baseline, 3-months, 6-months and 9-months from randomisation as shown in Figure 1.

The trial is recruiting participants with a diagnosis of schizophrenia or schizoaffective disorder with at least a mild level of depression, defined as a score of 14 or over on the Beck Depression Inventory II [17]. Recruitment to the trial began in May 2014 and is due to be competed in December 2015. Follow-up assessments are due to begin in August 2014 and to end in September 2016.

### Intervention

Positive memory training (PoMeT) is designed to enhance access to positive self-representations resulting in reduced levels of depression and increased self-esteem. It is based on a theoretical account of mood disorder which suggests that positive self representations are relatively dormant within individuals suffering from depression, at least in part due to infrequent activation. The first stage of therapy involves the patient identifying their core negative self-representation, such as being “worthless”. Within the first session the therapist moves towards the identification of personality characteristics which are inconsistent with being “worthless”. Homework involves writing as many specific examples as possible within which the patient has demonstrated these characteristics, e.g. being thanked for being a good friend. Subsequent sessions move towards working with one of these specific ‘positive memories’ in order to maximize the emotional impact. This includes the integrated use of imagery, posture and self-statements whilst ‘reliving’ this past positive event. It is important that the meaning of the positive event provides a direct challenge to the originally identified negative self-representation. A major component of the intervention protocol is daily practise of positive self-representation recall in between the weekly sessions. The final stages of the intervention involves the patient being trained to activate the positive self-representation at a time when an external environmental cue triggers the negative self-representation.

The intervention is manualised and highly structured. Whilst the original protocol adopted 8 sessions of one hour, on the advice of a service-user focus group our study protocol allows up to 12 sessions of one hour within the 3-month treatment period.

The treatment requires therapists to have a core mental health profession and a basic understanding of cognitive behaviour therapy. Within the current trial the intervention is delivered by a nurse who is an accredited cognitive behavioural therapist, a counselling psychologist and a clinical psychologist. Supervision is provided by team members with relevant expertise both in the intervention and patient group. Adherence to protocol is monitored through the use of a measure specifically designed for the current study and aimed to differentiate between the delivery of PoMeT and standard CBT or counselling.

### Treatment as usual (TAU)

Treatment as Usual (TAU) will be provided by the standard in-patient and out-patient services provided by the NHS. This will include neuroleptic medication and the possibility of other psychosocial interventions. All medication and service input received by both the PoMeT + TAU and the TAU groups will be documented during trial assessments.

### Inclusion and exclusion criteria

Potential participants must meet the inclusion criteria of (i) DSM-V diagnosis of schizophrenia of schizoaffective disorder (ii) at least a mild level of depression as measured by scoring 14 or more on the Beck Depression Inventory-II [17] (iii) aged between 18 and 65 (iv) have a sufficient understanding of spoken English to engage with assessments and clinical intervention (v) are able and willing to provide informed consent (vi) do no exhibit an organic impairment which is considered to be the primary diagnosis and (vii) do not exhibit a learning disability.

### Recruitment and randomisation

Recruitment will take place within two UK NHS Trusts, Berkshire Healthcare Foundation Trust and Southern Health Foundation Trust. Eligible participants are identified by trial research assistants, working in collaboration with care coordinators based within community mental health teams. Potentially eligible participants are invited to take part and provide informed consent. Once informed consent has been obtained trained researchers screen patient records to confirm eligibility and administer the Beck Depression Inventory in order to ascertain whether the patient is experiencing at least a mild level of depressed mood. If the patient is eligible the researcher continues with the baseline assessment battery. On completion of assessments the participant is randomised to either the PoMeT + TAU or the TAU group.

Randomisation is stratified by site and severity of depression (above and below a BDI-II score of 29, i.e. a severe level of depression) using randomised-permuted blocks. Group allocation is revealed to the participant, trial manager and trial therapists.

### Measures

#### Primary outcome measure

The primary outcome is current level of depressed mood as assessed by the Beck Depression Inventory- II [17].

#### Secondary outcome measures

Self-esteem is measured using the Roseneberg Self-Esteem Scale [18] which contains ten items measured on a four-point scale. The scale measures state self-esteem by asking the patient to reflect on their current feelings.

Psychotic Symptoms are assessed using two measures. The Positive and Negative Syndrome Scale (PANSS) [19] includes scales of positive symptoms, negative symptoms and general psychopathology and is used widely in schizophrenia research. Also, the Psychotic Symptom Rating Scale (PSYRATS) [16] is used to provide detailed information on the clinical characteristics of hallucinations and delusions, including distress and impact on functioning.

Anxiety is measured using the Generalised Anxiety Disorder Assessment (GAD7) [20] which includes seven items each describing anxiety symptoms, and endorsed on the basis of frequency. Functioning is measured using the Work and Social Adjustment scale [21]. Patient well-being is assessed using the Warwick-Edinburgh Mental Well-being Scale [22], a fourteen-item questionnaire which includes both hedonic and eudaimonic features which are rated on a five-point scale.

A semi-structured interview will be developed in consultation with service-user focus groups to explore the acceptability of the intervention. All participants who are allocated to the Positive Memory Training group will be invited to participate in an interview.

Patients’ health-related quality of life will be measured by the EuroQol EQ-5D [23], a generic, multi-attribute utility scale widely used for economic evaluations. In addition, patients will complete the ICECAP-A [24] and OxCAP-MH [25] instruments , both based on Sen’s capability approach that considers a broad concept of wellbeing including an individual’s ability to ‘do’ and ‘be’ the things that are important in life [26].

Primary informal carers will complete the EuroQol EQ-5D [23] and the Carer Experience Scale [27] via postal questionnaire to measure separately the likely impacts of PoMET on carers’ quality of life and wellbeing.

Costs will be assessed from a broad societal perspective using an amended version of the Client Service Receipt Inventory [28] and therapists’ records. These will include information on all PoMET treatment related costs, other health care resource use costs (including in-patient stays, outpatient visits, community mental health service contacts, primary care contacts, and medications), social care costs, and broader societal impacts (including costs to the patients and their families and lost productivity costs). Costs will be calculated using UK national-level unit costs.

Assessments will be conducted at baseline, 3-months (end of treatment) and at 6-months and 9-months after randomisation. All measures will be completed at baseline, 3-month and 9-month assessment. The 6-month assessment includes only measures of depression, patient health status (including quality of life and wellbeing) and costs.

### Analysis

#### Power

A sample of 50 per group will have 80% power to detect the PoMeT + TAU group presenting with a BDI-II score of 7 or more lower than the TAU group at the 2.5% level of significance (two-tailed).

#### Planned analysis

All analyses will be carried out on an intention to treat principle. Outcome measures for the intervention and treatment as usual (control) groups will be compared using linear mixed-effects models. The dependant variables will be the outcome measures at post-intervention and follow-up. Outcome measures at baseline, group membership and time will be included as fixed explanatory variables. The models will also include random effects for patients to allow for the correlation between post-intervention and follow-up outcome measures. Additionally, the models will contain interaction between time and group to test whether the effects of the intervention and usual treatment vary between post-intervention and follow-up time points. Standard diagnostic evaluations of model assumptions will be conducted to ensure valid inference.

The economic evaluation will be a within trial cost-utility analysis comparing the cost-effectiveness of PoMET in comparison to TAU and will follow the current gold standard methods for economic analyses. Cost-effectiveness will be primarily measured in terms of cost per quality adjusted life year gained (QALY). Alternative analyses will explore the cost-effectiveness of PoMET using capability measures. Uncertainty in the base case results will be assessed using non-parametric methods and extensive sensitivity analyses [29].

#### Planned subgroup analysis

Analyses will be conducted so as to assess for the role of self-esteem mediating any observed change in levels of depression within the treatment group. Also, self-esteem and depression will be assessed as potential mediators of change within the severity of psychotic symptoms within the treatment group.

## Discussion

Although depression is highly prevalent within individuals diagnosed with schizophrenia, there are no current evidence based interventions for the treatment of this specific co-morbid presentation. Relatively short-term structured interventions are easier to disseminate within health services, and therefore the Positive Memory Training protocol being adopted within the current trial has the potential to be both widely available and cost-effective. It is also envisaged that as the dominant focus of the intervention is on positive past experiences, that rates of patient engagement will be high.
